# Binge Eating (BE) and Obesity: Brain Activity and Psychological Measures before and after Roux-En-Y Gastric Bypass (RYGB)

**DOI:** 10.3390/nu15173808

**Published:** 2023-08-31

**Authors:** Shaunte Baboumian, Lauren Puma, Charles Swencionis, Nerys M. Astbury, Jennifer Ho, Spiro P. Pantazatos, Allan Geliebter

**Affiliations:** 1 Department of Psychiatry, Icahn School of Medicine at Mount Sinai, Mount Sinai Morningside, 1111 Amsterdam Ave, New York, NY 10025, USA; 2 Ferkauf Graduate School of Psychology, Yeshiva University, 500 West 185th Street, New York, NY 10033, USA; 3 Nuffield Department of Primary Care Health Sciences, Medical Sciences Division, University of Oxford, Oxford OX2 6GG, UK; 4 Molecular Imaging and Neuropathology Division, New York State Psychiatric Institute, Department of Psychiatry, Columbia University Irving Medical Center, 1051 Riverside Dr, New York, NY 10032, USA

**Keywords:** fMRI, bariatric surgery, region of interest (ROI) analysis, binge eating, individuals with obesity

## Abstract

Brain activity in response to food cues following Roux-En-Y Gastric Bypass (RYGB) in binge eating (BE) or non-binge eating (NB) individuals is understudied. Here, 15 RYGB (8 BE; 7 NB) and 13 no treatment (NT) (7 BE; 6 NB) women with obesity underwent fMRI imaging while viewing high and low energy density food (HEF and LEF, respectively) and non-food (NF) visual cues. A region of interest (ROI) analysis compared BE participants to NB participants in those undergoing RYGB surgery pre-surgery and 4 months post. Results were corrected for multiple comparisons using liberal (*p* < 0.006 uncorrected) and stringent (*p* < 0.05 FDR corrected) thresholds. Four months following RYGB (vs. no treatment (NT) control), both BE and NB participants showed greater reductions in blood oxygen level-dependent (BOLD) signals (a proxy of local brain activity) in the dorsomedial prefrontal cortex in response to HEF (vs. LEF) cues (*p* < 0.006). BE (vs. NB) participants showed greater increases in the precuneus (*p* < 0.006) and thalamic regions (*p* < 0.05 corrected) to food (vs. NF). For RYGB (vs. NT) participants, BE participants, but not NB participants, showed lower BOLD signal in the middle occipital gyrus (*p* < 0.006), whilst NB participants, but not BE participants, showed lower signal in inferior frontal gyrus (*p* < 0.006) in response to HEF (vs. LEF). Results suggest distinct neural mechanisms of RGYB in BE and may help lead to improved clinical treatments.

## 1. Introduction

Currently, the most effective treatment for obesity is bariatric surgery [1]. The elective nature of bariatric surgery frequently permits patients to select their preferred surgery type. Roux-en-Y gastric bypass (RYGB) is a popular operation and one of the most effective of the bariatric surgery procedures [1]. RYGB creates a 30-mL gastric pouch to reduce gastric capacity. The pouch is then anastomosed to a roux-limb through which gastric contents flow, bypassing most of the stomach [2]. Neuroimaging studies pre- and post-surgery have shown decreases in brain activation in mesolimbic reward pathway regions in response to high energy density food cues in fasted and fed states [1,3,4,5,6,7]. Additionally, post-RYGB, there is reduced desire for high energy density foods (HEF) vs. low energy density foods (LEF), which may be related to post-surgical reductions in mesolimbic activity in response to HEF vs. LEF cues [3,4,8,9].

Binge eating disorder (BED) is characterized by recurrent binge eating episodes (consumption of an unusually large amount of food in a short period of time with loss of control) [10]. According to the *Diagnostic and Statistical Manual of Mental Disorders-5* (DSM-5), binge frequency should average at least once a week for three consecutive months without compensatory behaviors [10]. Binge episodes are associated with marked distress and three or more characteristic psychological and behavioral symptoms. BED is found in much greater proportion among individuals with obesity and increases with greater degrees of obesity, such as in those undergoing bariatric surgery [11,12,13,14].

Studies have shown that individuals with obesity have heightened brain activity in response to food cues in brain regions associated with reward, motivation, emotions, memory, and decision-making and in brain areas involving attention and control [15,16]. We previously reported increased activity of the ventral tegmental area (VTA) (a dopaminergic reward region) in individuals with obesity vs. those of normal weight in response to HEF cues [15,17] and increased activity of the frontal premotor area in response to HEF cues in those with obesity and binge eating (BE) (vs. those with obesity without BE and those who are lean with and without binge eating) [18,19], which may reflect concurrent motor planning about ingesting palatable binge-type foods.

Dopaminergic signaling within the mesocorticolimbic reward pathway is associated with the hedonic value of reward stimuli, such as palatable food [20], which contributes to the initiation of motivated behavior (e.g., consumption) [20]. Schienle et al. (2009) found heightened medial orbitofrontal cortex (OFC) reactivity to HEF cues in individuals with BED as well as a positive correlation between medial OFC activity and self-reported reward responsiveness and elevated sensitivity for primary rewards [20,21]. We also previously found increased activity in the dorsal anterior cingulate cortex, potentially reflecting reward-based decision-making in response to HEF cues in women with BE vs. those without [17,22].

Although there is phenotypic overlap between obesity and BED, individuals with both obesity and BED represent a special subset of the obese population [23,24], experiencing more problematic eating behavior and more psychological problems, including higher rates of depression, lower self-esteem, and lower quality of life [23,25,26,27], as well as distinctive brain activation patterns [18,22,28]. Those with BED binge eat in part to regulate negative emotions [23,29], and negative emotional eating is positively correlated with the presence and severity of binge eating [29]. The tendency to eat more in response to negative emotions may contribute to weight gain and obesity [30,31]. Heightened psychological drive for food has also been linked to binge eating and obesity [32,33].

The prevalence of BED in bariatric surgery candidates ranges between 6–64% [34]. However, following surgery, likely due to the surgically-reduced stomach capacity, binge eating is eliminated in the short term [1,35], although some patients continue to exhibit loss of control with smaller amounts of food. Given the evidence for binge eating-related differences in neural responses to food stimuli in regions associated with reward and inhibition [15,36], we hypothesized that those with obesity and binge eating (BE) would show different brain activity patterns in response to food cues following bariatric surgery (primary outcome).

In this study, we used fMRI to observe brain activity patterns in response to food vs. non-food (NF) and HEF vs. LEF cues in individuals with obesity, with and without BE, pre- and 4 months post-surgery vs. those not treated (NT). We focused on nine previously defined ROIs with peak activation pre- and post-RYGB: the precuneus, dorsal cingulate, dorsomedial prefrontal cortex (dmPFC), anterior cingulate, thalamus, middle occipital gyrus, dorsolateral prefrontal cortex (dlPFC), precentral gyrus, and inferior frontal gyrus (Appendix B). Given that individuals with BE show greater responses to food cues [15], we expected that they would show a larger decrease in activation post-surgery vs. the NB group. Similarly, we expected they would show greater reductions in emotional eating and drive for food.

## 2. Methods

This study is part of a larger R01 study on functional brain imaging and appetite-related hormones pre- and post-obesity surgery, in a subset of only females to preclude heterogeneity due to gender effects. The protocol was reviewed and approved by the Mount Sinai Institutional Review Board and the IRB at Columbia University Medical Centre. All participants provided written informed consent before they were enrolled in the study. The study was prospectively registered on clinicaltrials.gov as NCT01590914. Participants were compensated ($500) and reimbursed for travel expenses.

The participants were healthy Class III (BMI > 40 kg/m^2^) women with obesity, including 15 who opted for RYGB surgery and 13 BMI-matched women who did not undergo treatment (NT). Surgery participants were recruited from patients at Mount Sinai Morningside Hospital in New York City (formerly St. Luke’s Hospital) [37]. Participants were eligible once the decision was made to undergo RYGB [38,39,40]. RYGB is a frequently used surgery option at our center. As described in Ames et al. 2017, treatment type is a shared decision and discussion between the provider and the patient, focused on metabolic disease severity, surgical and psychological risk factors, and patient preference [41]. NT participants were recruited via local newspaper and Craigslist ads. At baseline, all participants completed the Questionnaire of Eating and Weight Patterns Revised (QEWP-R) and the Eating Disorders Examination Questionnaire (EDE-Q), and participants were assessed according to the DSM-5. Participants were classified as BE if they endorsed overeating with loss of control and either met full criteria or subthreshold criteria, i.e., lower binge frequency and/or fewer binge eating characteristics. Participants were classified as NB if they did not meet either full criteria or sub-threshold criteria for BE.

Only women were included in the analysis to compare findings with our previous RYGB studies with women [4,5,6] and because of the sex-based differences in neural activation in response to food cues [42]. The NT group served as a control, including for possible habituation and practice effects of repeated fMRI protocols. They were also requested to maintain (within 10%) their starting weight throughout the 4-month follow-up period. Participant characteristics are shown in Table 1. A follow-up period of 4 months was used because surgery patients return to eating solid food about 5 weeks after surgery and have poor follow-up rates over a longer term [43]. After the analyses were completed, we discovered that one of the RYGB participants had actually undergone sleeve gastrectomy surgery instead of RYGB. We opted to keep the participant included in the analysis given previous reports that find little to no differences in fMRI neural activation between the two surgeries in response to HEF and LEF visual stimuli [3,8].

### 2.1. Inclusion/Exclusion Criteria

Potential participants underwent phone screening using the following inclusion criteria: severe obesity (BMI = 40–50 kg/m^2^), 18–65 years old, candidacy for RYGB or indication of not planning to undergo any weight loss interventions for the duration of the study (NT), relatively good health, i.e., absence of diabetes, heart disease, cancer, sleep apnea, and normal/well-controlled blood pressure. Exclusions were: 10% weight fluctuation in the previous three months; pregnancy (urine test), lactation, planning to become pregnant in next 18 months, or <1 year postpartum; smoking cessation in the previous three months or smoking more than five cigarettes per week; known claustrophobia for an fMRI enclosure; metal implants, non-removable metallic dental retainers, or pacemakers; consumption of four or more alcoholic drinks per day; recreational drug use in the previous six months; history of anorexia nervosa; history of hospitalization for a psychiatric condition; history of alcohol or drug dependence; previous bariatric surgery. Eligible participants provided informed consent approved by the hospital IRB and underwent a physical exam and a fasting blood draw, and urine pregnancy test.

### 2.2. Research Procedures

Participants underwent fMRI approximately 1 month before and 4 months after bariatric surgery. The NT group time interval was matched. Each procedure followed an overnight fast (except for water) past 8 pm the previous night. Just before the fast, participants were asked to consume 500-mL of a standard liquid meal (Glytrol). Glytrol is a mixed macronutrient test meal with a low glycemic index and was chosen because it poses minimal risk for postsurgical dumping syndrome (1 kcal/mL, 18% protein, 40% carbohydrate, 42% fat).

At baseline and 4 months post-surgery, body weight was measured with a Tanita scale employing bioelectrical impedance analysis (BIA) to measure body fat [44]. The participants fasted overnight and ingested 250 mL of Glytrol (about 1 h prior to the scan) for meal standardization and completed the Emotional Appetite Questionnaire (EMAQ) and Power of Food Scale (PFS) questionnaires. BE status was reassessed at 4 months using the QEWP-R and the Obesity Disorders Eating Questionnaire (ODE-Q), which also includes some modified questions pertaining to bariatric surgery in place of the EDE-Q [45]. For consistency, both RYGB and NT participants completed the ODE-Q. Participants were again classified as BE or NB (DSM-5).

### 2.3. fMRI Protocol

fMRI scans were conducted in the morning between 9–11 am, keeping the same time for a given participant. Scans were collected using a 1.5 GE Tesla twin-speed scanner with supine quadrature RF head coil. Three-plane localization was used to verify head position. Functional T2*-weighted images with a gradient echo pulse sequence (echo time = 60 ms, repetition time = 4 s, flip angle = 60°) were obtained. The visual stimuli were presented to participants in the scanner via goggles, in six runs. Each run was made up of 10 stimuli of the same type (HEF, LEF, NF) and repeated twice with different stimuli. The HEF stimuli included cakes, ice cream, and fast foods. The LEF stimuli consisted of fruits and vegetables. The NF stimuli consisted of basic office supplies, such as tape, staplers, and rubber bands. All HEF stimuli had an energy density of at least 3.5 kcal/g, and all LEF stimuli had an energy density less than 1 kcal/g. All the stimuli were used in our previous fMRI studies.

Stimulus types were presented in a pseudorandom order, with nonconsecutive runs of each type. Ten stimuli were presented for four seconds each (40 s), with a 52-s pre-stimulus baseline (crosshairs) and a 40-s post-stimulus baseline (crosshairs), resulting in a total of two minutes and 12 s per run. During each run, 36 whole-brain scans were taken, each consisting of 25 contiguous slices (4 mm thick), parallel to the AC/PC line (19 × 19 cm^2^ view, 128 × 128 matrix size, 1.5 × 1.5 mm^2^ in plane resolution). The first three scans of each run (12 s) were discarded to attain magnetic equilibration.

Nine ROIs were explored based on the ROIs with the highest-contrast t values (peak activation) for HEF > LEF pre > post-RYGB for activity in response to HEF, LEF, and NF stimuli at baseline, post-surgery and for changes from pre- to post-surgery [4] (Matlab 8.1 (MathWorks, Natick, MA, USA) and SPM8 (UCL Queen Square Institute of Neurology, London, UK)). ROIs consisted of 10 mm radius spheres centered at the Montreal Neurological Institute (MNI) coordinates identified in the above study.

### 2.4. Assessments

Emotional Appetite Questionnaire (EMAQ). The EMAQ assesses emotional eating in response to both positive and negative emotions and situations [46,47,48]. The EMAQ contains 22 questions, with 14 emotions and 8 situations (i.e., as compared to usual, do you eat more or less when you are sad, bored, confident etc. and when under pressure, after a heated argument etc.). For each emotion, the Likert scale ranges from 1 to 9, with “much less” (1), “the same” (5), and “much more” (9). There are also “non-applicable” and “don’t know” options. Mean scores are calculated for positive emotions and situations and negative emotions and situations [46]. The instrument has a high Cronbach alpha and good reliability [46,47,49].

Power of Food Scale (PFS). The PFS assesses the impact of current food-abundant environments, including appetite-related thoughts, feelings, and motivations. Individuals with obesity and BED scored higher than those with obesity or normal weight without BED [50,51]. The PFS assesses food liking and appetitive drive for palatable foods at three levels of food proximity: (1) food available (Abstract subscale), (2) food present (Presence subscale), and (3) food tasted (Pleasure subscale) [50]. Of the items, 21 are measured on a 5-point Likert scale, ranging from “don’t agree at all” to “strongly agree”. The PFS has been shown to have a high Cronbach alpha and good reliability [52]. Questionnaire data were analyzed using the *Statistical Package for the Social Sciences* (SPSS), 21st and 22nd Editions.

### 2.5. Behavioral Data Analysis

*Baseline characteristics.* One-way ANOVA was conducted on baseline characteristics, including age, BMI, weight (kg), and body fat percentage to compare between treatment groups (RYGB vs. NT) and between BE vs. NB status.

*Change in weight.* One-way ANOVA was also conducted for weight change, from baseline to 4 months post-treatment between treatment groups (RYGB vs. NT).

*Psychological Measures.* MANCOVA was conducted at baseline to determine the effect of binge eating status on the two emotional eating measures (i.e., negative and positive emotional eating) and three PFS measures (i.e., Abstract, Presence, and Pleasure) with baseline BMI as a covariate. ANCOVA was conducted for the 4-month outcome psychological measures, with the baseline score entered as a covariate. F tests and post hoc *t* tests were used to examine the effect of RYGB (vs. NT) on emotional eating and power of food in BE and NB groups. Treatment and binge eating status were entered as fixed factors. Two-tailed *p* ≤ 0.05 was considered significant.

### 2.6. fMRI Imaging Analysis

*Power analysis.* Required sample sizes were estimated with G*POWER 3.1 for power = 0.80, with 2-tailed α = 0.05, and effect size Cohen’s d. It was predicted that from baseline to 4 months post-surgery, there would be a similar reduction in brain activation in the BE and NB groups. The closest study is by Ochner et al. (2011), with fMRI presurgery and 1-month post-RYGB, using a similar stimulus paradigm, showing reduced activation post-RYGB when comparing HEF and LEF cues in the ventral striatum, with t = 2.17, d = 1.02, which would require *n* = 17 for comparing RYGB pre to post [4]. Since changes in brain activation at 4 months post-surgery was expected to be similar to 1 month post-surgery, there should be adequate power with *n* = 28.

*ROI definition.* Regions-of-interest (ROIs) were generated using 10 mm spheres around MNI coordinates that we previously reported for nine ROIs: the inferior frontal gyrus, precuneus, dorsal cingulate, dorsomedial prefrontal cortex, anterior cingulate, thalamus, middle occipital gyrus, dorsolateral prefrontal cortex, and precentral gyrus [4].

*fMRI image processing.* The BOLD imaging data were analyzed using Statistical Parametric Mapping, 8th Edition. Prior to statistical analyses, the realigned T2*-weighted volumes were slice-time corrected, spatially transformed to a standardized brain (Montreal Neurologic Institute) and smoothed with an 8-mm full-width half-maximum Gaussian kernel. We then applied a single-subject (1st-level) fixed-effects model on both sessions (baseline and 4 months post) followed by a group random effects (2nd-level) model. First-level regressors were created by convolving the onset of each condition with the canonical hemodynamic response function (HRF) with duration of 40 s. The following contrasts were created from the resulting estimated parameters for visual categories:

(1) Food > Non-Food (NF)
(2) High Energy Density Food (HEF) > Low Energy Density Food (LEF) at (a) baseline [Timepoint 1 (T1)], (b) 4 months post [Timepoint 2 (T2)], and (c) Timepoint 1 > Timepoint 2 (T1 > T2).

Food stimuli consisted of high and low energy-density food and non-food consisted of office supplies.

*Hypothesis-driven contrasts:* The hypotheses for these four contrasts apply to eight regions of interest, including the precuneus, dorsal cingulate, dorsomedial prefrontal cortex, anterior cingulate, thalamus, middle occipital gyrus, dorsolateral prefrontal cortex, and precentral gyrus, while findings were expected to go in the opposite direction for the inferior frontal gyrus: (1) At T1: HEF > LEF, BE > NB, (i.e., BE compared to NB will have greater brain activation in response to HEF vs. LEF cues); (2) At T2: HEF > LEF, BE > NB, (i.e., at 4 months post-surgery or NT, BE compared to NB will have greater brain activation in response to HEF vs. LEF cues); (3) At T2: HEF > LEF, NT > RYGB, BE > NB (i.e., at 4 months post, BE participants who have RYGB vs. BE participants who do not undergo surgical treatment will have less brain activation in response to HEF vs. LEF cues); (4) T1 > T2, HEF > LEF, NT > RYGB (i.e., from baseline to 4 months post-surgery or NT, both BE and NB participants who have RYGB will have a similar reduction in brain activation in response to HEF vs. LEF cues, as compared to relatively low changes in brain activation in NT.

*Second level model.* The contrasts of parameter estimates from the 1st level (within-subject) models (T1, T2, T1 > T2, Food > NF, and HEF > LEF) were then passed on to 2nd level multiple regression analyses, which consisted of predictor variables for experimental group (BE vs. NB; RYGB vs. NT) and BMI as a nuisance covariate. Main comparisons included overall effects of experimental group at baseline (T1), 4 months post-surgery (T2), and baseline > post (T1 > T2) contrasts as well as two-way interactions between experimental (BE and NB) and intervention (RYGB and NT) groups at baseline (T1), post (T2), and baseline > post (T1 > T2) contrasts (see above for the specific contrasts tested).

Mean BOLD signal was extracted for each ROI and imported into SPSS for quality control and statistical inference. Data from the five outcome measures were assessed for outliers, normality, and homogeneity of variance for the four groups (RYGB BE, RYGB NB, NT BE, and NT NB) at baseline and 4 months post-surgery. Boxplots were used to identify outliers and extreme scores. Normality was assessed in three ways: (1) graphs (i.e., histograms, P-P plots, Q-Q plots), (2) numerically (i.e., skewness, kurtosis), and (3) significance tests (i.e., Kolmogorov–Smirnov test). Homogeneity of variance was assessed with Levene’s test. Given that there were outliers, non-normal data, and unequal variances in the dataset, bootstrapping was used to analyze the data. One thousand bootstrap samples were performed [53] (p. 694), using a bias corrected and accelerated confidence interval. Test statistics for original, unsampled data are reported, but degrees of freedom are not, since accompanying *p*-values were estimated using nonparametric bootstrapping as described above.

*Thresholding.* Two significance thresholds were used: (a) *p* < 0.05 corrected, calculated based on controlling the false discovery rate (FDR) across all tests conducted [54], and (b) a less stringent exploratory threshold (*p* < 0.006 uncorrected) calculated by dividing one over the total number of tests conducted, 19 contrasts × 9 ROIs = 171 (1/171 = 0.006), which indicates that there would be only one false positive by chance at this threshold [55]. All findings that survive *p* < 0.006 uncorrected are reported, while those surviving *p* < 0.05 FDR corrected are indicated as well.

## 3. Results

### 3.1. Participant Characteristics

There were no significant differences in age, BMI, body weight, or body fat percentage between the BE and NB groups (Table 2). Body weight and BMI were greater in the RYGB group compared with the NT group, but the groups did not differ in age (Table 2). At baseline, eight RYGB and seven NT participants were classified as BE, and seven RYGB and six NT individuals were classified as NB, according to the QEWP-R and the EDE-Q. All those with BE endorsed overeating with loss of control. The BE group included participants who met full DSM-5 binge eating criteria as well as subthreshold (i.e., reported less than one binge episode per week on average or engaged in fewer than three of the five associated binge eating behaviors). The BE group scored higher on negative emotional eating (5.7 ± 1.3 SD, *p* < 0.05) and similar positive emotional eating (4.6 ± 1.4, ns) compared to NB individuals (4.0 ± 1.6; 4.2 ± 1.5, respectively) compared with the NB group. BE subjects also scored higher on the PFS Abstract (19.9 ± 8.3, *p* < 0.05), PFS Presence (22.3 ± 7.2, *p* < 0.05), and PFS Pleasure (22.5 ± 7.6, *p* < 0.05) than NB participants (PFS Abstract 11.2 ± 3.6; Presence 14.5 ± 3.6; Pleasure 14.3 ± 4.3).

### 3.2. 4 Months Follow-Up (T2)

At 4 months post-surgery, the RYGB group had a lower score for PFS presence (*p* = 0.01) and pleasure of food (*p* = 0.006) compared with the NT group, but there were no differences in PFS Abstract (*p* = 0.15). RYGB (vs. NT) reported less negative emotional eating at 4 months post as indicated by EMAQ Negative (*p* = 0.05), but no difference for EMAQ Positive (*p* = 0.40). At 4 months post, there were no significant differences between RYGB BE participants and RYGB NB participants for PFS Presence and PFS Pleasure scores.

### 3.3. Baseline to 4 Months (T1 > T2)

There was a significant difference in weight change from baseline to 4 months between groups, *F*(1, 24) = 276.9, *p* < 0.001. The RYGB group lost 24.3 kg ± 4.0, and the NT group lost 1.2 kg ± 3.0. There were no significant differences in weight loss between RYGB BE participants (24.7 kg) and RYGB NB participants (23.8 kg), *p* = 0.69. There was no interaction effect in the treatment × BE group on PFS Abstract (*p* = 0.267), EMAQ Negative (*p* = 0.996), and EMAQ Positive (*p* = 0.507).

### 3.4. fMRI Outcomes

#### 3.4.1. Baseline (T1)

**Figure 1:**
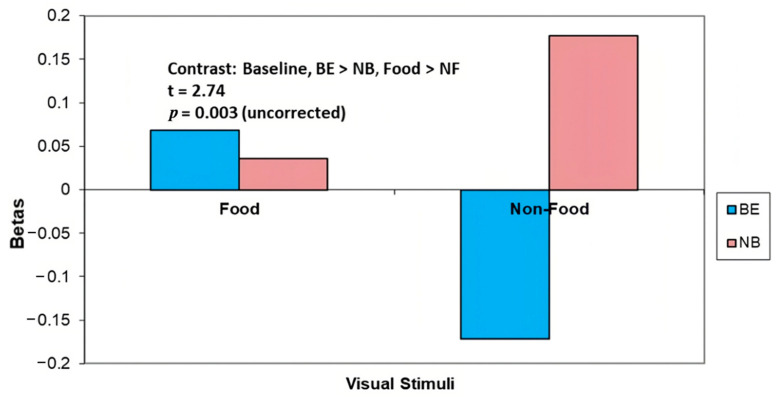
At baseline, binge eaters (BE) showed greater activation (vs. non-binge eaters, NB) in precuneus in response to Food vs. Non-Food (NF) cues.

*Food* vs. *NF:* There was greater precuneus brain activity in the BE group compared with the NB group in response to Food vs. NF cues at baseline (t = 2.74, *p* = 0.003 uncorrected, *p* = 0.12 corrected) (Figure 1, Table 2). Precuneus activation in BE (vs. NB) participants in response to NF was both lower and negative (i.e., lower activity relative to the crosshairs control).

*HEF* vs. *LEF cues:* No results reached our level of significance (*p* < 0.006 uncorrected).

#### 3.4.2. 4 Months Post (T2)

**Figure 2:**
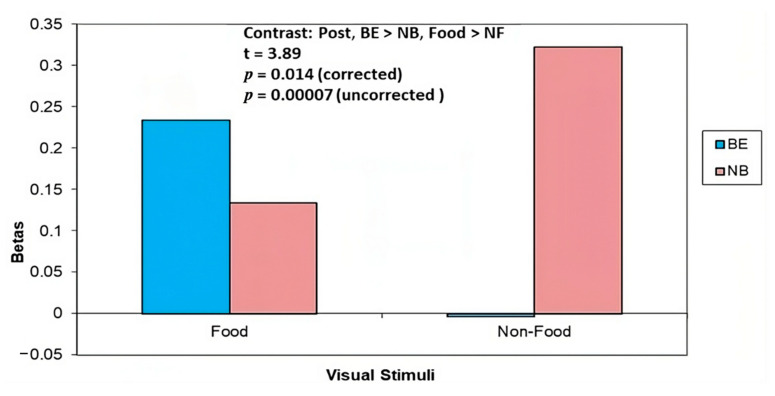
At 4 months post-surgery or no treatment, binge eaters (BE) showed greater activation (vs. non-binge eaters, NB) in the thalamus in response to Food vs. Non-Food cues.

**Figure 3:**
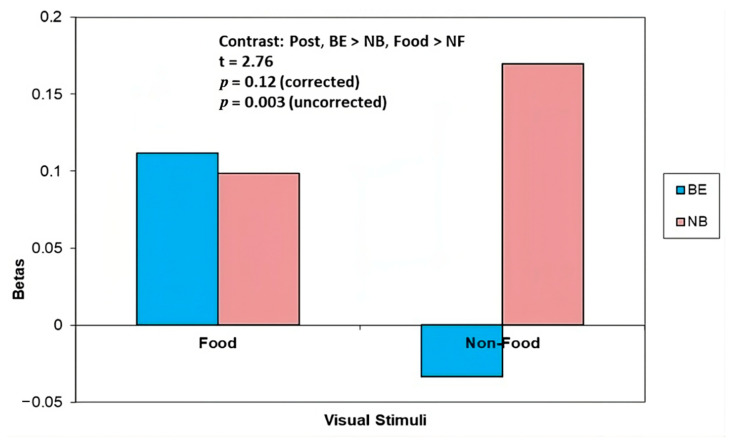
At 4 months post, binge eaters (BE) showed greater activation (vs. non-binge eaters, NB) in precuneus in response to Food vs. Non-Food (NF) cues.

*Food* vs. *NF:* There was greater brain activity in the thalamus in the BE group in response to Food (vs. NF) cues, whilst in the NB group there was greater brain activity in the thalamus in response to NF (vs. Food) cues (BE > NB, Food > NF t = 3.89, *p* = 0.00007 uncorrected, *p* = 0.014 corrected) (Figure 2, Table 3). There was also greater brain activity in the precuneus region in the BE group compared with the NB group in response to Food (vs. NF) cues (t = 2.76, *p* = 0.003 uncorrected) (Figure 3, Table 3). Similar to baseline, at 4 month post-surgery follow-up (T2), brain activity in the precuneus region in the BE group in response to NF cues was reduced relative to the crosshairs.

**Figure 4:**
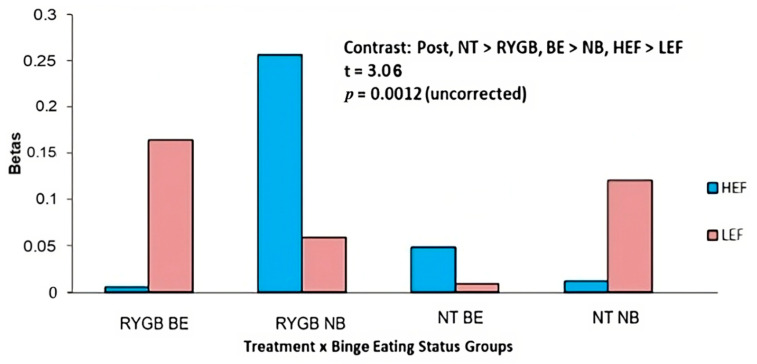
At 4 months post-surgery, there was a two-way interaction between treatment and binge eating status groups in the middle occipital gyrus in response to HEF (vs. LEF) cues. Binge eaters (BE) showed greater activation (vs. non-binge eaters, NB) in middle occipital gyrus in response to Food vs. Non-Food (NF) cues. The RYGB BE participants (vs. RYGB NB, NT BE, and NT NB participants) had the lowest middle occipital gyrus activation in response to HEF (vs. LEF) cues, whilst the RYGB NB participants (vs. RYGB BE, NT BE, and NT NB participants) had the highest middle occipital gyrus activation in response to HEF (vs. LEF) cues.

*HEF* vs. *LEF cues:* There was an interaction between treatment x experimental group (t = 3.06, *p* = 0.0012 uncorrected) for brain activity in the middle occipital gyrus (Figure 4, Table 3). Plots of parameter estimate contrasts revealed that RYGB BE participants (vs. RYGB NB, NT BE, and NT NB participants) had the lowest middle occipital gyrus brain activity in response to HEF. The brain activity increased in RYGB BE participants in response to LEF, whilst there was no significant change in brain activity (from crosshairs) in response to HEF. In comparison, the RYGB NB participants had the highest brain activity in the middle occipital gyrus in response to HEF cues.

**Figure 5:**
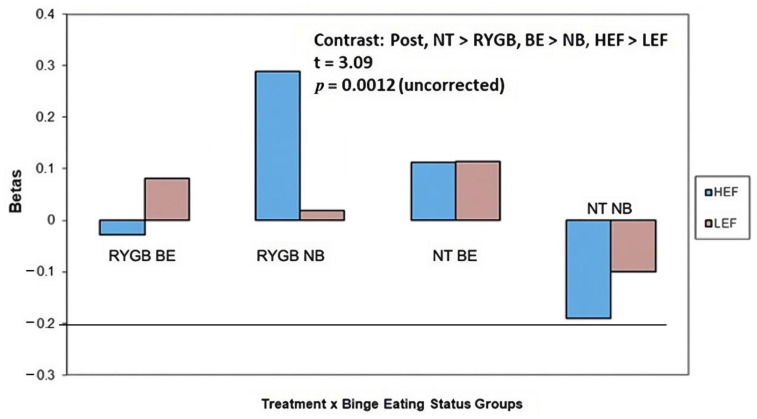
At 4 months post-surgery, there was a two-way interaction between the treatment and binge eating status groups in the inferior frontal gyrus in response to HEF (vs. LEF) cues. The RYGB NB group (vs. RYGB BE, NT BE, and NT NB participants) had the highest inferior frontal gyrus activation in response to HEF (vs. LEF) cues, whilst the NT NB participants (vs. RYGB BE, RYGB NB, and NT BE participants) had the lowest inferior frontal gyrus activation in response to HEF vs. LEF (which had a negative activation).

There was also an interaction between the treatment and experimental groups (4 months post-surgery: NT > RYGB, BE > NB, HEF > LEF t = 3.09, *p* = 0.003 uncorrected) for brain activity in the inferior frontal gyrus (Figure 5, Table 3). The RYGB NB group had the highest inferior frontal gyrus brain activity in response to HEF cues. In comparison, the NT NB group had the lowest inferior frontal gyrus brain activity in response to HEF. The RYGB BE group had the second lowest inferior frontal gyrus brain activity in response to HEF cues.

#### 3.4.3. Change in Activation from Baseline to 4 Months Post (T1 > T2)

*Food* vs. *NF:* No results survived *p* < 0.006 uncorrected.

**Figure 6:**
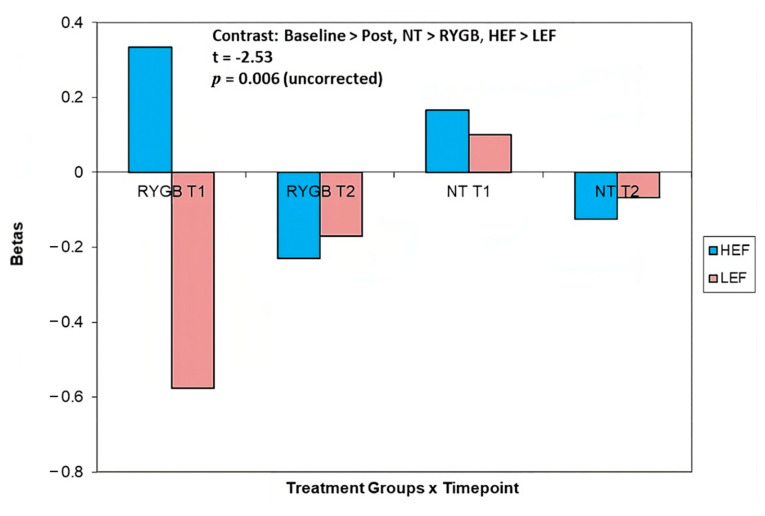
There was a two-way interaction between treatment and binge eating status groups in pre to post surgery change in dorsomedial prefrontal cortex (dmPFC) responses to HEF (vs. LEF) cues. From baseline to 4 months post-surgery, RYGB (BE and NB participants) had reduced dmPFC activation in response to HEF (vs. LEF) food cues. RYGB showed an increase (though still negative), whilst NT showed a decrease, in response to LEF from baseline to 4 months post.

*HEF* vs. *LEF:* There was an interaction between experimental group and treatment (Baseline > Post, NT > RYGB, HEF > LEF, t = −2.53, *p* < 0.006 uncorrected) for pre- > post-surgery changes in activation in the dmPFC (Figure 6, Table 3). From baseline to 4 months post-surgery, RYGB (BE and NB participants) had reduced dmPFC activation in response to HEF (vs. LEF) food cues. RYGB showed an increase (though still negative), whilst NT showed a decrease, in response to LEF from baseline to 4 months post (Figure 6).

## 4. Discussion

Greater BOLD signal, a proxy measure of brain activity in the precuneus in BE participants relative to NB participants in response to food vs. NF at baseline and post-surgery suggests that food cues were more salient and relevant for BE participants. This finding is consistent with the obesity “neural phenotype” as individuals with obesity (vs. of normal weight) show greater neural responses to food stimuli in attention and memory regions [16]. Similarly, at 4 months post-surgery, BE (vs. NB) participants had more thalamic brain activity in response to food cues than NB participants. In BE participants, the food stimuli may be a more salient bottom-up sensory stimulus that is processed via the thalamic relay to visual attention cortical areas. These findings suggest that post-RYGB, binge eaters show different neural response to food despite binge eating behavior no longer being present after surgery. Imaging and behavioral studies demonstrate marked changes in appetite, salience, and food preferences, including addiction transference syndrome [9,56].

At 4 months post-surgery, RYGB BE participants compared to NT BE participants had significantly less brain activity middle occipital gyrus in response to HEF cues, suggesting that HEF (vs. LEF) cues are less salient in RYGB BE participants as a result of surgery. Additionally, RYGB BE participants compared to NT BE participants had significantly less activation in the inferior frontal gyrus, a region implicated in top-down inhibition of HEF food cues [57], suggesting less regulatory responses are recruited, possibly because of reduced bottom-up salience of HEF cues following RYGB ([5].

There was a significant reduction in activation of the dmPFC following RYGB, in both the BE and NB groups in response to HEF vs. LEF cues. The dmPFC is part of the mesocortical reward pathway [58], and has been associated with emotional eating and reward-based decision making [59,60,61,62]. Contrast plots suggest that NT also showed a (non-significant) reduction in dmPFC activation in response to HEF v. LEF cues, and that RYGB had a significantly greater reduction. In addition, relative to NT, RYGB showed an increase in activation in response to LEF (vs. HEF) food cues, suggesting activity in this region is associated with an increase in liking for LEF foods in RYGB.

### 4.1. Questionnaire Findings

At baseline, BE participants compared to NB participants had significantly greater negative emotional eating (EMAQ) and psychological drive for food (PFS) scores. All RYGB BE participants stopped binge eating after surgery, likely due to their surgically reduced gastric capacity. Post-RYGB compared to NT had less negative emotional eating and a lower psychological drive (presence and pleasure) for food in both BE and NB participants. Thus, surgery reduced responsivity to presence and pleasure of food in both BE and NB participants.

The findings suggest evidence for a “neural BE phenotype” indicated by greater baseline brain activation in the precuneus and thalamus in response to Food (vs. NF) cues, which differs from the obesity NB phenotype. The BE phenotype appears to persist post-surgery since RYGB (vs. NT) BE participants continue to have significantly greater thalamic and precuneus activation in response to Food (vs. NF) cues at 4 months post. Nevertheless, following RYGB, both BE and NB participants showed similar weight loss, altered activation in the dmPFC and inferior frontal gyrus in response to HEF (vs. LEF) cues, and reduced negative emotional eating and psychological drive for food.

While BE (vs. NB) participants showed more negative emotional eating and greater psychological drive for food at baseline, at 4 months post-surgery there were no longer differences in emotional eating and psychological drive for food between BE and NB participants; however, RYGB BE participants had significantly lower presence of food and pleasure of food scores than NT BE participants, indicating that surgery had a corrective effect on BE participants.

### 4.2. Strengths and Limitations

This study has several strengths: it is a longitudinal study, which prospectively examined BE and NB participants with obesity from pre- to post-RYGB in comparison to a non-treated BE and NB control group. This helps differentiate between obese and binge eating neural phenotypes. By scanning participants following a meal, neural responsivity to HEF vs. LEF cues were examined postprandially, when binge episodes usually occur. In addition, ROI analyses, as opposed to whole brain analyses, were performed, which better control for Type I error by limiting the number of statistical tests. Moreover, this study administered measures to assess binge eating, emotional eating, and psychological drive for food before and after RYGB, which sheds light on how bariatric surgery impacts eating behavior. Some limitations include a relatively small sample size and short follow-up period. Intervention groups were not randomly assigned, given the ethical considerations of elective surgery. The generalizability of this study is limited to individuals with severe obesity. Ethnicity was predominantly African American and Hispanic females from an urban neighborhood, groups largely overrepresented in bariatric surgery candidates.

## 5. Conclusions

This study findings suggest that binge eaters show different neural responsivity to binge foods than non-binge eaters following bariatric surgery. Binge eaters (vs. NB participants) also appear to show reduced middle occipital and inferior frontal gyrus activation to binge foods following surgery. Binge eaters seem to show greater precuneus activation (at baseline and post-surgery) and thalamic (post-surgery) activation in response to food (vs. non-food) cues, suggesting that food is a more salient “bottom-up” stimulus in BE participants. BE participants appear to devote more cortical processing resources to these food cues relative to NB participants, even 4 months after surgery, although they report reductions in negative emotional eating and psychological drive for food.

Besides significant weight loss, RYGB in BE participants may lead to changes in how they perceive and respond to HEF (vs. LEF) cues, as suggested by reduced middle occipital gyrus and inferior frontal gyrus activation in RYGB BE participants (vs. NT BE participants). These changes suggest that HEF cues are less salient and elicit less response inhibition (inferior frontal gyrus) to control eating behavior in post-RYGB BE participants. Surgery also may lead to a reduction in dmPFC responses to HEF cues, which may be involved in less consumption of HEF vs. LEF after surgery. With regards to eating behavior, post-RYGB BE participants reported no binge eating, less negative emotional eating, and lower psychological drive for food compared to NT BE participants. They also showed decreased responsivity to the presence and pleasure of food (PFS subscales) from pre-surgery to 4-months post-surgery. Weight loss was comparable for BE and NB participants at 4 months post, suggesting that preoperative BE status does not hinder postoperative weight loss in the short-term.

Currently, the best clinical treatment outcomes for binge eating behavior require strong compliance, a large time commitment, and other substantial resources. Studies show that long-term cognitive behavioral therapy and interpersonal psychotherapy, often combined with pharmacological intervention, is the best course of treatment [63]. Given the relationship between binge eating behavior and obesity, further elucidation of the neural mechanisms of bariatric surgery changes to appetite and eating behavior may have an impact on treatment options. There is also potential to develop novel treatment strategies focusing on neural mechanisms that circumvent the need for malabsorptive restrictive surgeries.

## Figures and Tables

**Table 1:** Ethnic and Racial % and (*n*) by Treatment Group.

Ethnic	RYGB ^a^	NT
Hispanic or Latino	60 (9)	69.2 (9)
Not Hispanic or Latino	40 (6)	31.8 (4)
Racial		
American Indian/Alaska Native	0	0
Asian	0	0
Native Hawaiian or Other Pacific Islander	0	0
Black or African American	40 (6)	61.5 (8)
White	0	7.7 (1)
Other	33.3 (5)	15.4 (2)
Unknown	26.7 (4)	0
More Than One	0	15.4 (2)

Roux-en-Y Gastric Bypass (RYGB), Non-Treatment (NT); ^a^ One of the participants underwent sleeve gastrectomy in lieu of RYGB surgery. NOTE: Ethnic and racial categories are in accordance with the National Institute of Health guidelines (NIH, 2015). The “Other”, “Unknown”, and “More Than One” categories were also included.

**Table 2:** Baseline Characteristics of Participants with Binge Eating (BE) and Non-Binge Eating (NB) and Self-Report Scores (M ± SD).

	BE	NB	RYGB	NT
*n*	15	13	15	13
Age (years)	38.5 ± 12.5	32.3 ± 8.8	37.0 ± 10.4	33.9 ± 12.3
Body mass index (kg/m^2^)	42.8 ± 4.0	43.3 ± 3.7	44.7 ± 3.9	41.1 ± 2.8
Weight (kg)	116.5 ± 15.3	115.7 ± 13.7	121.8 ± 13.4	109.5 ± 12.8
Body fat (%) ^a^	50.3 ± 3.4	49.2 ± 3.8	50.9 ± 2.9	48.5 ± 3.9
EMAQ Negative ^b^	5.7 ± 1.3 *	4.0 ± 1.6 *	4.9 ± 2.1	4.9 ± 0.8
EMAQ Positive ^b^	4.6 ± 1.4	4.2 ± 1.5	3.9 ± 1.7	5.0 ± 0.7
PFS Abstract ^b^	19.9 ± 8.3 *	11.2 ± 3.6 *	14.5 ± 7.5	17.5 ± 8.2
PFS Presence ^b^	22.3 ± 7.2 *	14.5 ± 3.6 *	16.9 ± 6.5	20.1 ± 7.1
PFS Pleasure ^b^	22.5 ± 7.6 *	14.3 ± 4.3 *	16.8 ± 7.6	20.9 ± 6.9

* Significant differences, *p* < 0.05. ^a^ Missing data for one participant in each group. ^b^ Points as scored by the questionnaires’ validated scales. Binge eating participants (BE), non-binge eating participants (NB), Emotional Appetite Questionnaire (EMAQ) Score Range: 1–9, Power of Food Scale (PFS) Sum Score Range: 21–105, Power of Food Scale (PFS) Subscale Score Range per subscale: 7–35.

**Table 3:** Regions of Interest (ROI) t-values for the four main hypothesized contrasts (see methods).

ROI Coordinates (x, y, z)	ROI Label	Baseline BE > NB Food > NF	Post BE > NB Food > NF	Post NT > RYGB BE > NB HEF > LEF	Baseline > Post NT > RYGB HEF > LEF
−20, −64, 30	Precuneus	2.74 *	2.76 *	2.3	−1.71
32, 36, 6	Inferior Frontal Gyrus	0.38	2.17	3.09 *	−1.14
−6, 2, 36	Dorsal Cingulate	2.0	1.98	2.0	−1.84
8, 58, 16	Dorsomedial Prefrontal Cortex	1.17	−0.18	1.27	−2.53 *^a^
20, 38, 22	Anterior Cingulate	2.26	1.46	2.13	−1.26
−14, −22, 4	Thalamus	0.81	3.89 **	1.94	−1.81
34, −70, 6	Middle Occipital Gyrus	1.16	0.41	3.06 *	−1.65
28, 22, 32	Dorsolateral Prefrontal Cortex	1.31	1.55	1.74	−1.49
−62, −12, 38	Precentral Gyrus	1.43	0.76	1.05	−1.86

* Significant at *p* < 0.006 uncorrected. ** Significant at *p* < 0.05 corrected. Baseline and 4 months post-surgery BMIs were entered as covariates. ^a^ From baseline to 4 months post-surgery, the RYGB and NT groups had reduced activation in this region in response to High (vs. Low Energy Density) Food, whereas RYGB had an increase and NT had a decrease in response to Low (vs. High Energy Density) Food.

**Figure A1:**
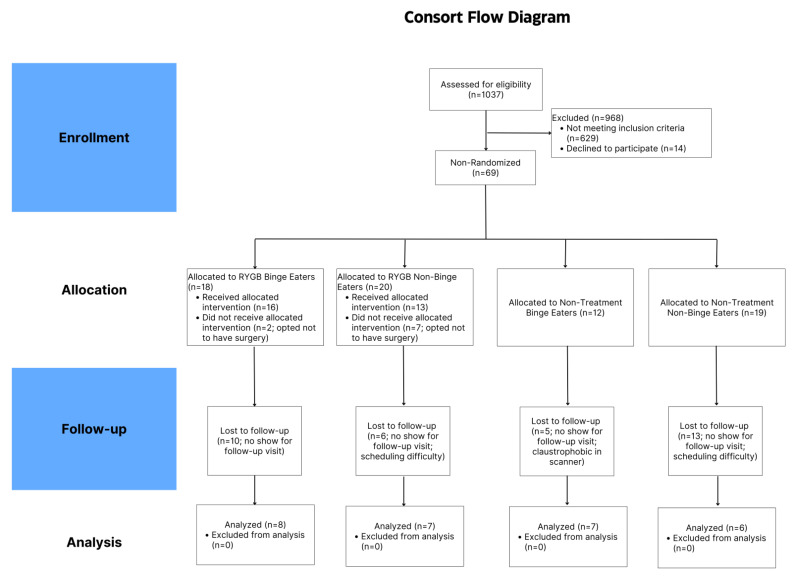
Flow diagram describing participant eligibility and inclusion.

**Figure A2:**
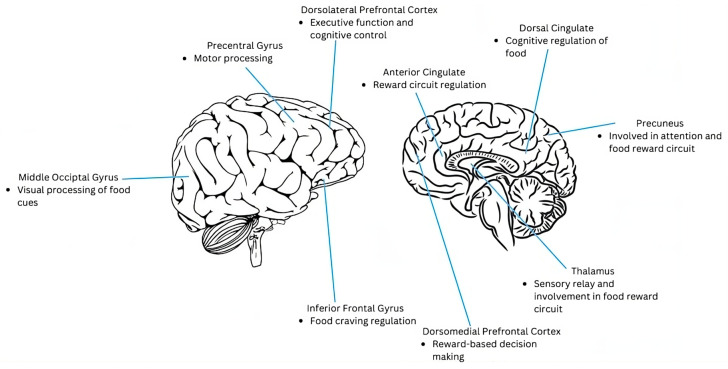
Diagram of brain with ROIs labeled, corresponding to Table 3.

## Data Availability

The data reported in this manuscript may contain potentially sensitive and identifiable information. Requests for secondary use of the data should be made directly to Allan Geliebter (allan.geliebter@mountsinai.org) or Shaunte Baboumian (shaunte.baboumian@mountsinai.org).
